# Management of proximal femur fractures in the elderly: current concepts and treatment options

**DOI:** 10.1186/s40001-021-00556-0

**Published:** 2021-08-04

**Authors:** H. Fischer, T. Maleitzke, C. Eder, S. Ahmad, U. Stöckle, K. F. Braun

**Affiliations:** 1 Department of Oral and Maxillofacial Surgery, Charité–Universitätsmedizin Berlin, Augustenburger Platz 1, 13353 Berlin, Germany; 2 Julius Wolff Institute, Berlin Institute of Health at Charité– Universitätsmedizin Berlin, Augustenburger Platz 1, 13353 Berlin, Germany; 3 Center for Musculoskeletal Surgery, Charité–Universitätsmedizin Berlin, Augustenburger Platz 1, 13353 Berlin, Germany; 4 BIH Biomedical Innovation Academy, BIH Charité Clinician Scientist Program, Berlin Institute of Health at Charité–Universitätsmedizin Berlin, Charitéplatz 1, 10117 Berlin, Germany; 5 Klinik Und Poliklinik Für Unfallchirurgie, Klinikum Rechts Der Isar der TU München, Ismaninger Street 22, 81675 München, Germany

**Keywords:** Garden classification, Frailty, Surgical management, Delirium prevention

## Abstract

As one of the leading causes of elderly patients’ hospitalisation, proximal femur fractures (PFFs) will present an increasing socioeconomic problem in the near future. This is a result of the demographic change that is expressed by the increasing proportion of elderly people in society. Peri-operative management must be handled attentively to avoid complications and decrease mortality rates. To deal with the exceptional needs of the elderly, the development of orthogeriatric centres to support orthogeriatric co-management is mandatory. Adequate pain medication, balanced fluid management, delirium prevention and the operative treatment choice based on comorbidities, individual demands and biological rather than chronological age, all deserve particular attention to improve patients’ outcomes. The operative management of intertrochanteric and subtrochanteric fractures favours intramedullary nailing. For femoral neck fractures, the Garden classification is used to differentiate between non-displaced and displaced fractures. Osteosynthesis is suitable for biologically young patients with non-dislocated fractures, whereas total hip arthroplasty and hemiarthroplasty are the main options for biologically old patients and displaced fractures. In bedridden patients, osteosynthesis might be an option to establish transferability from bed to chair and the restroom. Postoperatively, the patients benefit from early mobilisation and early geriatric care. During the COVID-19 pandemic, prolonged time until surgery and thus an increased rate of complications took a toll on frail patients with PFFs. This review aims to offer surgical guidelines for the treatment of PFFs in the elderly with a focus on pitfalls and challenges particularly relevant to frail patients.

## Introduction

The majority of proximal femur fractures (PFFs) affects the elderly as more than three quarters of PFFs occur in patients over the age of 75 in Germany [1]. While around 1.3 million hip fractures were reported globally in 1990 [2], the number is estimated to range between 7.3 and 21.3 million by 2050 [2].

For elderly patients, a PFF often represents a life-changing event, stripping patients of their already potentially impaired self-sustainability. Within 1 year after a hip fracture, only 40–60% of elderly patients regain their pre-fracture level of mobility and ability to perform daily living activities [3].

Comorbidities are high in patients with PFFs, with 50% of PFFs occurring in people with pre-existing nursing care needs [4]. A geriatric patient is defined as a patient above the age of 80 or a patient with typical geriatric multimorbidity in combination with an age of > 70 years [5].

Around 25–50% of people aged 85 and older are considered to be frail [6], meaning three or more of the following factors apply according to the definition of Fried et al.:

1. Unintentional weight loss;
2. Low grip strength;
3. Self-report of exhaustion;
4. Slow walking speed;
5. Low physical activity level [7].

Frailty describes a state of increased vulnerability to stressors, mostly due to a lack of resources [6]. Even a small event (e.g., minor infections like a urinary tract infection or minor surgery) may result in a striking and disproportional deterioration of the individual’s health status, due to the low resolution of homeostasis [6].

PFFs in frail patients are associated with a pronounced risk of cardiovascular, pulmonary, thrombotic, infectious, or bleeding complications [8] with further surgical delay increasing the risk of mortality [9].

Ideally, operative treatment should take place within the first 24 h [10]. Surgery after more than 24 h raises the chance for peri-operative complications such as pulmonary embolism, pneumonia, deep vein thrombosis, urinary tract infections and pressure ulcers. If surgery is delayed for more than 48 h, the mortality risk rises significantly. Patients operated within 48 h show a 20% lower risk of dying within the next year, and especially patients with comorbidities benefit significantly from surgery within 24 h [9].

There is evidence for reduced in-hospital complication rates, shorter hospital stays and fewer readmissions, as well as lower disability and in-hospital mortality when implementing interdisciplinary geriatric care in trauma management [11].

The aim of this article is to provide a comprehensive review of crucial aspects in the treatment of PFFs in elderly patients and to point out how to avoid complications in the peri-operative and postoperative periods.

Delay of operative treatment increases complications and mortality

## Anatomy of the femoral neck

In the hip joint, the almost spherical femoral head articulates with the hollow sphere of the facies lunata of the acetabulum. The articular cavity's surface takes up only 50% of the femoral head’s surface [12]. The femoral neck connects the femoral head with the shaft, forming an angle of approximately 127° [13], while its radiological outline shows compressive and tensile trabeculae, that characteristically form the ward triangle as a zone of low trabecular density [12]. For vertical reinforcement of the trabecular bone, the calcar femorale provides an essential contributor to stability [14]. Thus, a correct reduction of the calcar femorale is a key factor in the operative treatment of PFFs.

With age, the trabecular structure degenerates [15] and, concomitantly, reinforcements like the calcar femorale lose structural integrity. It was hypothesized that the neck-shaft-angle increases with age [16], yet data on more than 8000 neck-shaft-angles showed no significant differences between the age groups [17, 13].

Low energy falls, which become more frequent with age, are the leading cause of hip fractures. During such falls, compressive stress is applied to the femoral neck’s superolateral cortex, being considered the main mechanism of injury of PFFs [18]. Osteoporosis, loss of dense trabecular networks, an increased diameter and a thinner cortex of the femoral neck enhance buckling susceptibility [10, 19].

**Fig. 1:**
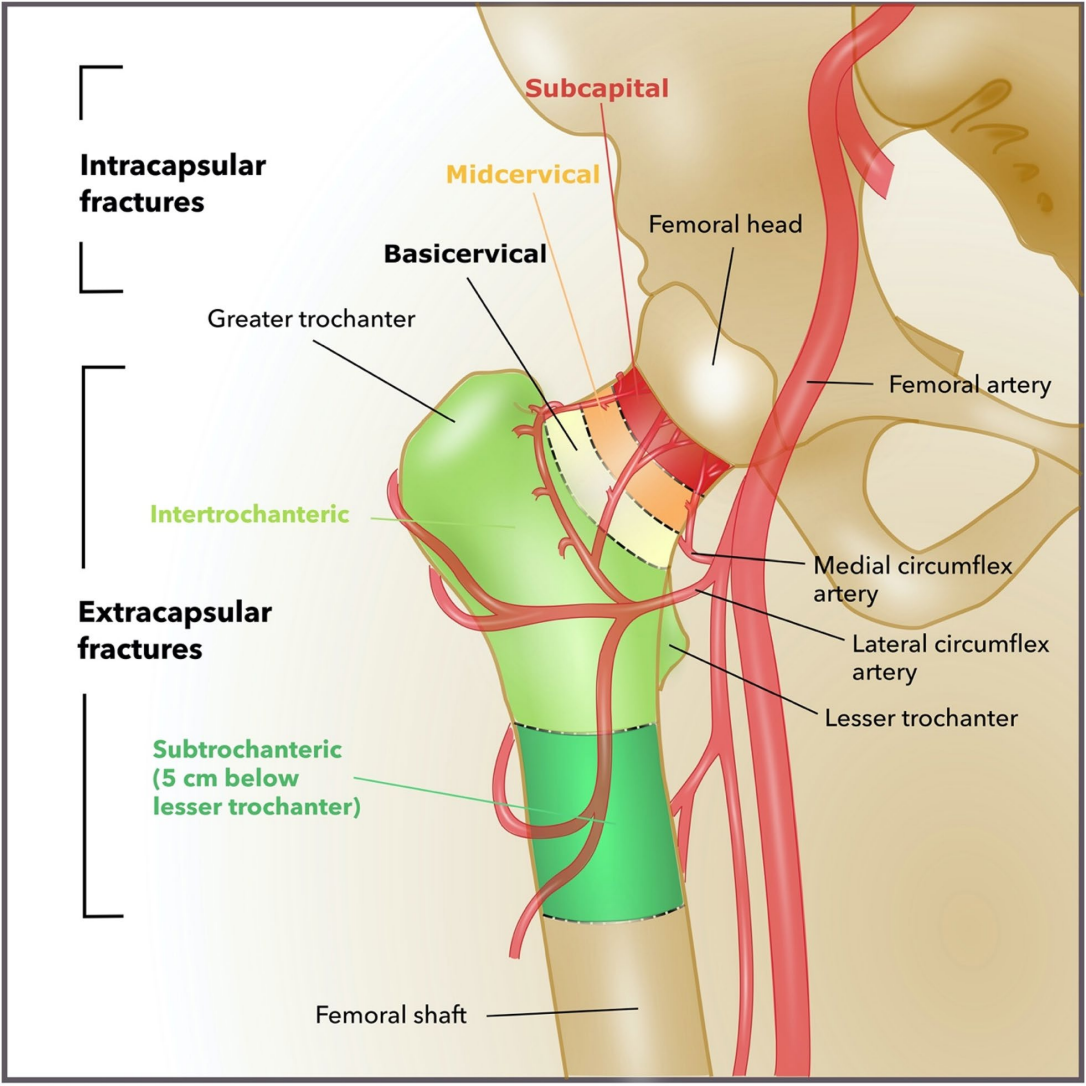
Bony and vascular anatomy of the proximal femur (adapted from [8])

Bone healing is dependent on the femoral head's vascular supply which might easily be disrupted by fracture dislocation or increased intracapsular pressure, and cellular coverage of the femoral head, which deteriorates with age, thus limiting osteoprogenitor cell influx following a femoral neck fracture. In adults, only 20% of the femoral neck’s surface is covered by cellular periosteum [20]. The femoral head receives its primary blood supply from the superior, anterior, and inferior retinacular arteries arising from the deep branch of the medial circumflex femoral artery as well as the round ligament arteries [21] (Fig. 1).

**Fig. 2:**
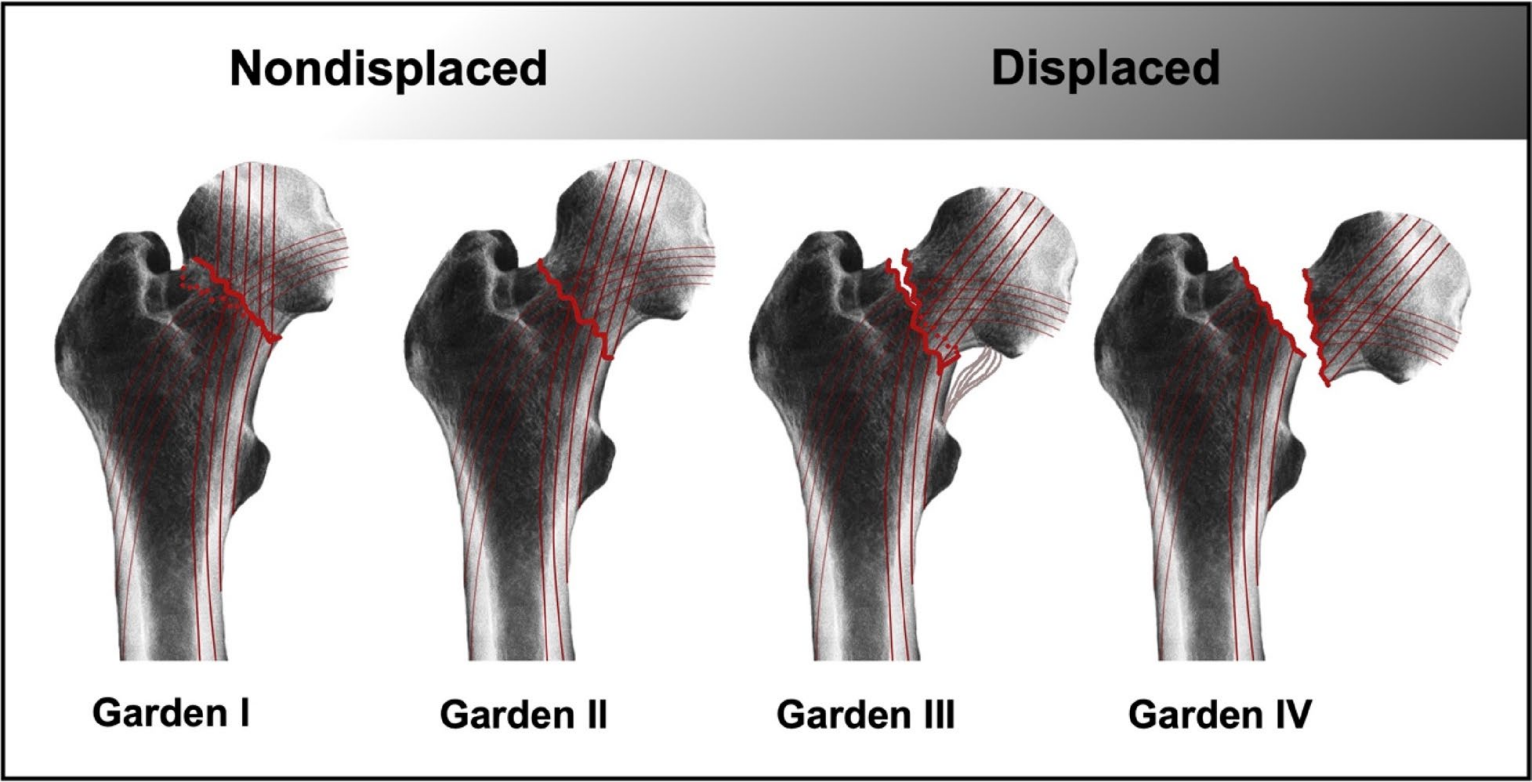
The Garden classification of non-displaced (Garden type I and II) and displaced (Garden type III and IV) femoral neck fractures. Incomplete or impacted fractures, including a valgus dislocation, are classified as type I. If neither impaction nor dislocation occurs, the fracture is classified as type II. Type III refers to a dislocated fracture with existing bony contact in the calcar femoris region, including the retinacula of Weitbrecht being still intact [77]. Type IV indicates a complete disassociation of the femoral head from capsule and vessels. A higher dislocation grade is associated with a higher probability of disruption of the femoral neck’s blood supply

The development of a posttraumatic femoral head necrosis is highly correlated with disrupted retinacular arteries, which pose the main blood supply for the femoral head [22]. In Garden Type IV fractures, all retinacular arteries appear disrupted as a result of gross dislocation [21]. The retinacula of Weitbrecht are intraarticular synovial plicae protecting the retinacular arteries within [23]. Among the anatomical variances, the medial retinaculum is constantly present [23], extending from the base of the lesser trochanter to the edge of the acetabular cartilage (Fig. 2) [23].

The poor vascular supply and a limited regenerative potential of the femur neck’s periosteum may cause impaired bone regeneration

## Classification of femoral neck fractures

PFFs are divided into intracapsular and extracapsular femoral neck fractures, including intertrochanteric and subtrochanteric fractures (Fig. 1). Depending on their location, femoral neck fractures are identified as sub-capital, mid-cervical, and basicervical fractures. Especially in the elderly, the mid-cervical femoral fracture is the most common by far, with a frequency of over 86% [24].

There are three common classifications for femoral neck fractures: The Garden, the Pauwels and the AO classification. First published by R.S. Garden in 1961, the Garden classification is the one most widely used. Femur neck fractures are classified by the fracture displacement based on an ap radiogram into non-displaced (Garden type I and II) and displaced fractures (Garden type III and IV). Garden type I describes an incomplete or impacted fracture, Garden type II a complete fracture without displacement, Garden type III a complete fracture with partial displacement, and Garden type IV a complete fracture with full displacement [25] (Fig. 2).

The Garden classification has only a fair inter-observer reliability when all four types are assessed, but a moderate to substantial one if fractures are only classified as undisplaced or displaced [26]. Fracture displacement correlates with interruption of the vascular supply, as described above; therefore, Garden classification relates to the risk of femoral head necrosis. Due to the disrupted blood supply to the femoral head [21], Garden type IV fractures are not suitable for osteosynthesis. However, if the fracture line is located at the very basis of the femoral neck, it decreases the risk of femoral head necrosis regardless of dislocation, because the fracture might be lateral to the vascular supply.

The Pauwels classification concentrates on the biomechanical forces adding pressure on the fracture line. Type I describes a dominating compression force, with a fracture line of up to 30° to the horizontal plane. In type II, shearing stress is present; the fracture line lies between 30° and 50° [27]. Shearing stress has a possible negative impact on bone healing [28]. In the third type with a fracture line above 50°, shearing stress is predominant, leading to fracture displacement [27]. In the inter-observer reliability, the Pauwels classification shows only weak reliability and reproducibility [26].

**Fig. 3:**
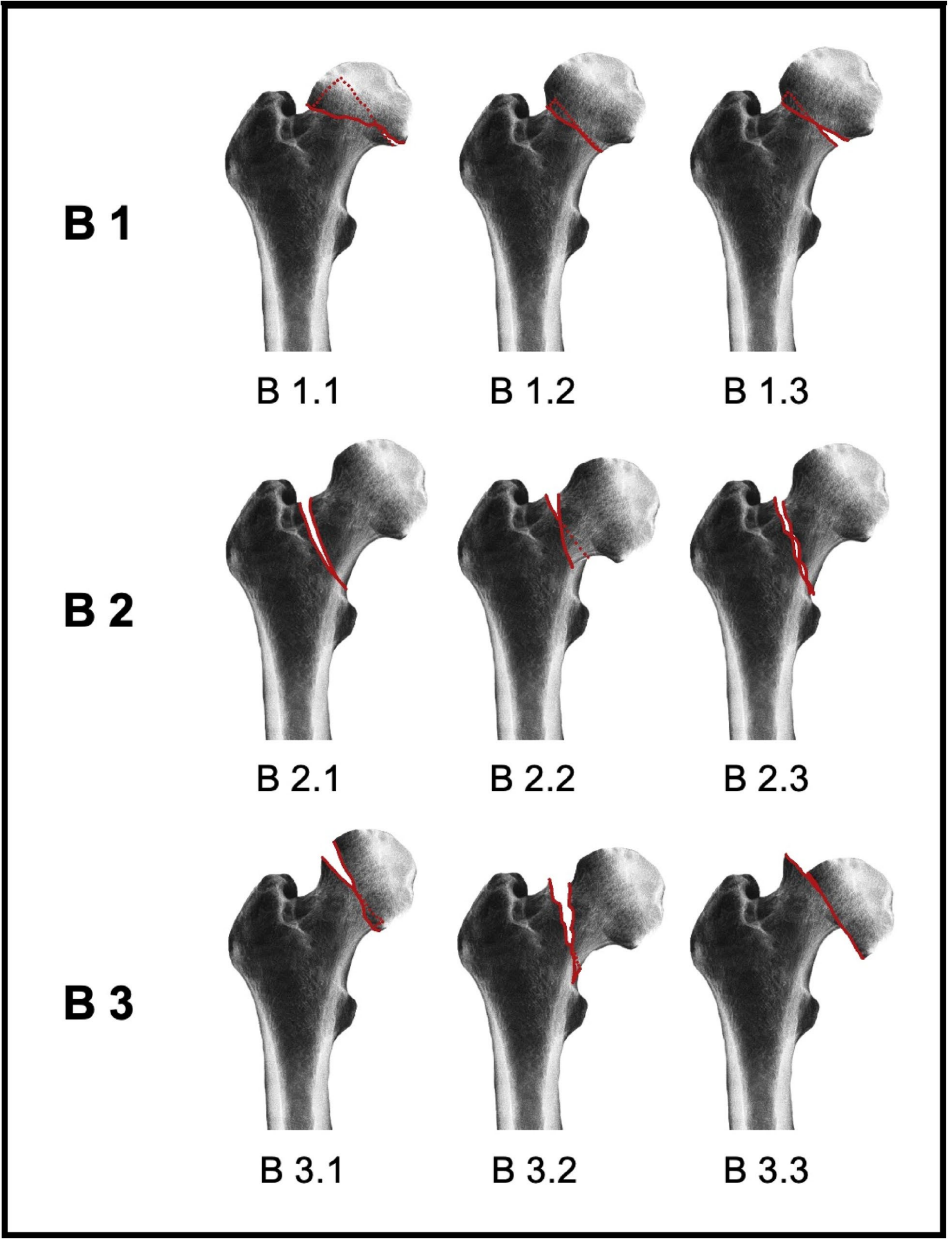
AO classification of femoral neck fractures. AO 31-B1 includes impacted fractures. With decreasing impaction from grade 1 to grade 3, B2 consists of a larger femoral head fragment with a fracture line increasing in slope from grade 1 to grade 3, and B3 describes a small head fragment with increasing dislocation and instability with increasing grade

As the most complex classification, the AO classification combines the fracture level, the degree of displacement, and the angle of the fracture line (Fig. 3). Because of its complexity, the AO classification serves mainly for academic purposes.

The Garden classification describes the risk of necrosis of the femoral head

## Peri-operative management

Frailty fractures are classified as fractures in the absence of adequate trauma or a fall from standing height or less with hip fractures represent the most common frailty fracture types [29].

A comprehensive geriatric assessment helps to identify treatable geriatric conditions to prevent complications in elderly patients. Evidence suggests that comprehensive geriatric assessment improves the outcome of people above the age of 65 with a hip fracture [30]. This can be done after surgery, since older people receiving comprehensive geriatric assessment are less likely to die and more likely to return to their previous environment.

For the radiological confirmation of the diagnosis of a PFF, an ap X-ray is sufficient. A second plane X-ray in most cases does not contain additional information but is often very painful for the patient. If available, a planning body for preoperative determination of the prosthesis size should be added if a prosthesis is needed. If an X-ray cannot confirm the diagnosis, but a hip fracture is highly suspected, it is recommended to perform a computed tomography (CT).

Sufficient pain management is mandatory and its importance needs to be expressed. Not only is it humane, but it is also an essential factor in the prevention of delirium [31]. In the peri-operative pain management of elderly patients, NSAIDs are not recommended. However, it is advised to offer non-NSAIDs such as paracetamol every 6 h unless contraindicated [32]. If no sufficient pain control is accomplished, i.v. or oral opioids can be titrated according to the patient’s constitution accompanied by a routine constipation prophylaxis [10].

If non-NSAIDs and opioids are not sufficient, femoral nerve blocks may be considered [32]. Guay et al. stated in a Cochrane review that there is moderate quality evidence for reducing pneumonia risk, decreased time to first mobilisation, and cost reduction in pain medication after single-shot blocks [33]. High-quality evidence suggests that a regional blockade reduces pain on movement within 30 min after block placement [33].

Routine laboratory tests should be performed on all patients, including complete blood count, inflammation markers, INR, partial thromboplastin time, and a basic metabolic profile [10]. As hip fracture patients tend to be dehydrated, i.v. hydration might be needed with the amount depending on clinical judgment. A flow rate of 100–200 ml/h for isotonic crystalloids is estimated to be safe [10]. Volume status needs to be monitored carefully, however, as many elderly patients have cardiac diseases, making them predisposed to heart failure triggered by volume overload [10].

With age, the incidence of urinary tract infections increases [34]. In addition to symptomatic urinary tract infections, asymptomatic bacteriuria is common among the elderly. It is estimated that asymptomatic bacteriuria is present in around 20% of healthy women over the age of 80 years [35].

The link between hardware infection and asymptomatic bacteriuria has been investigated, especially in the context of arthroplasty. Even though there is a correlation between an increased occurrence of prosthetic joint, superficial wound infections and the presence of asymptomatic bacteriuria, Zhang et al. showed in a systematic review that the incidence of postoperative infectious complications did not decrease when the asymptomatic bacteriuria is treated before arthroplasty [35]. In hip fracture patients, screening for urinary tract infections is recommended, although those should only be treated if symptomatic [10].

Proper pain management plays a crucial role in preventing complications

## Prevention of bleeding complications

Approximately 40% of elderly patients presenting with hip fracture are under anticoagulant or antiplatelet therapy [36]. Managing anticoagulants and antiplatelets requires close coordination with anaesthesiology. For patients receiving antiplatelet therapy, it is recommended to proceed with surgery directly rather than delaying surgery to restore platelet function [37]. In the case of dual antiplatelet therapy, spinal anaesthesia is contraindicated. The use of clopidogrel and particularly the combination of clopidogrel and aspirin might lead to increased peri-operative blood loss [38]. Nevertheless, it has also been shown that those patients can still safely undergo hip fracture surgery without delay [38].

INR values below 1.5 are desired in patients receiving vitamin K antagonists, including warfarin and phenprocoumon. This may be achieved by either waiting, i.v. vitamin K substitution or the administration of fresh frozen plasma before surgery [10]. A bridging strategy based on either treatment-dose subcutaneous low-molecular-weight heparin or intravenous unfractionated heparin should be considered for patients with mechanical valves, atrial fibrillation with recent history of stroke, deep vein thrombosis, or pulmonary embolism [39].

For the anti-Xa-agents (Apixaban, Edoxaban, Rivaroxaban), a plasma drug level of under 50 pg/ml is deemed safe for surgery [40]. If there is no possibility of measuring the plasma level, a gap of 24 h between the last dose and surgery should be considered.

For patients anti-coagulated with Dabigratran, there is a chance to determine the plasma level and use the direct anti-agent Idarucizumab for neutralisation [41]. The elimination of direct oral anticoagulants can be compromised, depending on renal and hepatic function (Table 1) [39].

**Table 1:** Anticoagulants and antiplatelets summarised [39]

Drug	Elimination half-life	Management	Acceptable to proceed with spinal
Aspirin	Irreversible effect on platelets	Proceed with surgery	Continue
Clopidogrel	Irreversible effect on platelets	Proceed with surgery, monitor for blood loss, consider platelet transfusion if concerns regarding bleeding	If anti-platelet monotherapy. General anesthesia if dual therapy
Ticagrelor	8–12 h	Proceed with surgery with general anaesthetic. Monitor for blood loss. Consider platelet transfusion if concerns regarding bleeding	5 days or post platelet transfusion at least 6 h post last dose
Warfarin	4–5 days	5 mg vitamin K i.v. and repeat INR after 4–6 h. This can be repeated or consider Beriplex for immediate reversal	If INR < 1.5
Apixaban	12 h	Surgery and anesthesia 24h after last dose if renal function is normal	2 half-lives/24 h after last dose if renal function is normal
Dabigatran	12–24 h	Surgery and anesthesia if thrombin time normal or idarucizumab for immediate reversal if thrombin time prolonged	If thrombin time normal or 30 min following idarucizumab infusion
Rivaroxaban	7–10 h	Surgery and anesthesia 24 h after last dose if renal function normal	2 half-lives/24 h after last dose if renal function normal
Low-molecular weight heparin sub-cutaneous prophylactic dose	3–7 h	Last dose 12 h pre-op	12 h
Low-molecular weight heparin sub-cutaneous treatment dose	3–7 h	Last dose 12–24 h pre-op. Monitor for blood loss	24 h
Unfractionated i.v. heparin	1–2 h	Stop i.v. heparin 2–4 h pre-op	4 h

Systemic administration of tranexamic acid can reduce blood loss and transfusion rates and can be used for control of bleeding in anti-coagulated patients. Yet, a recent meta-analysis showed that there is still a lack of evidence concerning the optimal regimen, timing, and dosage of tranexamic acid [42].

## Preventing delirium

Hypoactive delirium is present more often and frequently remains unrecognised in the elderly [43, 44]. It is associated with a higher rate of complications and mortality, so prevention plays a vital role [45].

For the screening of delirium in hospitalised older people, the 4AT is a sensitive and specific tool which is validated for hip fractures [46]. To determine mental status changes, it is important to establish a baseline status, for example using routine screening at admission.

For delirium prevention, multicomponent non-pharmacological approaches have been proven to be a good strategy [44]. Those approaches include early mobilisation, adequate hydration, sleep enhancement, orientation in time and place, hearing and vision optimisation as well as therapeutic activities such as reminiscence [44]. To additionally prevent delirium, a one face policy for visitors can be established alongside stress reduction and daytime activity to allow sleep at night in support of a normal night–day rhythm.

If delirium occurs, it is important to search for a possible reason in need of treatment, such as electrolyte derangements, metabolic derangements, infection, organ failure, pain, or anticholinergic load. With the help of an anticholinergic burden scale, e.g., the anticholinergic drug scale, inappropriate medication in elderly patients can be identified [47]. Using drugs with anticholinergic properties in the elderly increases the risk of delirium, cognitive impairment, falls, fractures, and mortality [48]. We recommend evaluating the indication and modalities of drug therapy for delirium together with a geriatrician (Table 2).

**Table 2:** Acceptable reasons for delaying surgery in hip fracture patients according to the guideline for the management of hip fractures 2020 by the Association of Anaesthetists [39, 49]

Acceptable	Unacceptable
Haemoglobin concentration < 8 g dL	Lack of facilities or theatre space
Plasma sodium concentration < 120 or > 150 mmol/l	Awaiting echocardiography
Potassium concentration < 2.8 or > 6.0 mmol/l	Unavailable surgical expertise
Uncontrolled diabetes	Minor electrolyte abnormalities
Uncontrolled or acute onset left ventricular failure	
Correctable cardiac arrhythmia with a ventricular rate > 120 min	
Chest infection with sepsis	
Reversible coagulopathy	

## Operative management

The goal for the treatment should always be the return to the previous level of activity and full weight bearing.

### Intertrochanteric and subtrochanteric fractures

Both in intertrochanteric and subtrochanteric fractures, the treatment of choice is intramedullary nailing as it decreases soft tissue damage and permits early weight bearing. For intertrochanteric fractures, the choice of implant depends on the stability of the fracture pattern defined by the lateral cortical wall [50]. Extramedullary devices like the sliding hip screw can be chosen if the lateral cortical wall is intact [50], making a thorough evaluation of the fracture pattern essential when an extramedullary device is considered.

In comparison to extramedullary devices such as the sliding hip screw, an intramedullary device is located closer to the vector of force line, equalising a shorter lever arm compared to extramedullary devices, thus giving intramedullary nails a biomechanical advantage [50].

Cheng and Sheng compared eight treatment options for intertrochanteric fractures [dynamic hip screw, compression hip, percutaneous compression plate, Medoff sliding plate, less invasive stabilisation system, gamma nail, proximal femoral nail, and proximal femoral nail anti-rotating (PFNA)] and identified PFNA as the preferable surgical method with fewer blood loss and high functional outcomes, according to the Harris hip score [51]. When using intramedullary nails, the use of a helical blade in comparison to a lag screw is associated with a higher rate of collapse of the neck-shaft angle and the concomitant dislocation of the screw (cut-out) in the femoral head [50].

Subtrochanteric fractures are a less common type of hip fracture. In subtrochanteric fractures, intramedullary nailing (long nail) is considered the gold standard, because it decreases operation time, fixation failure and length of hospital stay in comparison to extramedullary devices [52].

To reduce the risk of cut-out in screws and blades in osteoporotic bone, cement augmentation can be used in osteosynthesis, although it may risk thermal damage, osteonecrosis and cement leaking to the fracture region. In particular for PFNA, significantly improved rotational stability and pull-out resistance were shown biomechanically [53]. It was clearly demonstrated that cement augmentation enhances implant anchorage in osteoporotic bone [53]. A systematic review by Namdari et al. of the clinical results of cement augmentation indicated that the main benefits lie in improved radiographic parameters and lower complication rates when using cement augmentation. However, larger systematic studies are needed to further investigate the extent of the benefit [54].

### Femoral neck fractures

Femoral neck fractures can either be treated with osteosynthesis, total hip arthroplasty or hemiarthroplasty. In patients with more than one comorbidity above the age of 70, there is an 83% risk of secondary fracture dislocations when treated conservatively [55], making surgery the treatment of choice for elderly patients. When choosing the implant, two main aspects need to be kept in mind: older patients are less likely to follow weight-bearing restrictions [56], while, on the other hand, the indication for osteosynthesis needs to be carefully considered. Due to biomechanical aspects, according to Pauwels classification, any femoral neck fracture classified as type I or II is an indication for internal fixation. Due to the blood supply of the femoral head, femoral neck fractures classified as Garden type III and IV are, in most cases, not suitable for osteosynthesis. Dislocated femoral neck fractures are related to a high incidence of interrupted blood supply of the femoral head (as described above), and therefore, predisposed for fixation failure. Existing osteoporosis and age-related changes in bone structure might lead to an increased risk of non-unions in elderly patients [57]. Osteosynthesis is, therefore, suggested in either biologically young patients with non-dislocated fractures or as a salvage option, if the patient is bed-bound and operative therapy is only indicated for pain management.

Even though this review focuses on frail elderly patients, between 50 and 75% of elderly patients are not frail. It needs mentioning that in healthy and active patients, biological age should determine the choice of implant. The high functional requirements and lower biological age, in comparison to the chronological age of the so-called “golden-ager”, have led to a paradigm-shift towards total arthroplasty instead of hemiarthroplasty in healthy elderly patients [58].

There is good evidence that in hip arthroplasties, cemented implants lead to less postoperative pain and thereby better mobility [59]. A cemented femoral stem leads to a better fixation in osteoporotic bone [60]. Because no cortical press-fit needs to be achieved, only a reduced stem preparation is necessary, leaving a thicker cortical wall. This results in a potentially reduced periprosthetic fracture risk and lower loosening rates. In a German registry study, Konow et al. showed a two times higher risk of a periprosthetic femoral fracture in uncemented versus in cemented stems with a significantly increased risk for patients above the age of 60 when uncemented stems were used [61]. Therefore, a standard procedure should include a cemented shaft and, depending on the patient´s activity, a hemiarthroplasty or a total arthroplasty should be chosen. In active patients, a total arthroplasty is the implant of choice due to a better functionality and lower long-term reoperation rate in comparison to hemiarthroplasty. However, total hip arthroplasty might be linked to a higher rate of dislocation [60]. Procedure-related factors such as the surgical approach, the positioning of the components, the soft tissue tension, the surgeon´s experience, but also implant-related factors play a major role in the risk for dislocation following total hip arthroplasty [62]. Sarcopenia, the loss of proprioception, and an increased risk of falls are described as typical risk factors in the elderly [62]. For patients who are not able to follow precautions to lower the risk of dislocation, hemiarthroplasty might be the better option. For those with an elevated risk profile and suitable bone quality, a non-cemented shaft should be considered to lower the risk of bone cement implantation syndrome during the operation. Risk factors for suffering from bone cement implantation syndrome include impaired cardiopulmonary function, grade III and IV ASA levels, pre-existing pulmonary hypertension, poor pre-existing physical reserve and bony metastases [63].

The Dorr type and the cortical thickness are key factors in estimating the risk of an intraoperative fracture when placing the prosthesis and can thus help guiding the choice of the fixation method. The Dorr description of the proximal femoral morphology correlates with a low cortical thickness index [64]. In comparison to type A, Dorr type B and C indicate a higher risk of intraoperative fracture [64].

The advantages of hemiarthroplasty are a shorter operation time and a lower incidence of dislocation [58]. The HEALTH-Trial compared patients with displaced femoral neck fractures undergoing either total hip arthroplasty or hemiarthroplasty in a multicentre randomised controlled trial. No significant difference in the incidence of secondary procedures could be found, while functional endpoints according to the WOMAC score favoured total hip arthroplasty over hemiarthroplasty [65]. A slightly higher incidence of serious adverse effects could be seen in the group that underwent total hip replacement [65]. In biologically young patients, use of a hemiarthroplasty is linked to high rates of acetabular erosion and the need for conversion to total hip arthroplasty due to secondary osteoarthritis [66].

Accounting for only 1.8% of all PFFs, basicervical femoral neck fractures are quite uncommon [67]. The treatment options include both a cephalomedullary nail, a dynamic hip screw and cancellous screws. When the latter were used, a higher failure rate was observed [67]. Reviewing treatments and failures of basicervical femoral neck fractures, Yoo et al. stated that further research with a homogenous definition on treatment results or fixation failure are needed to perform a meta-analysis for clear recommendations [67].

**Fig. 4:**
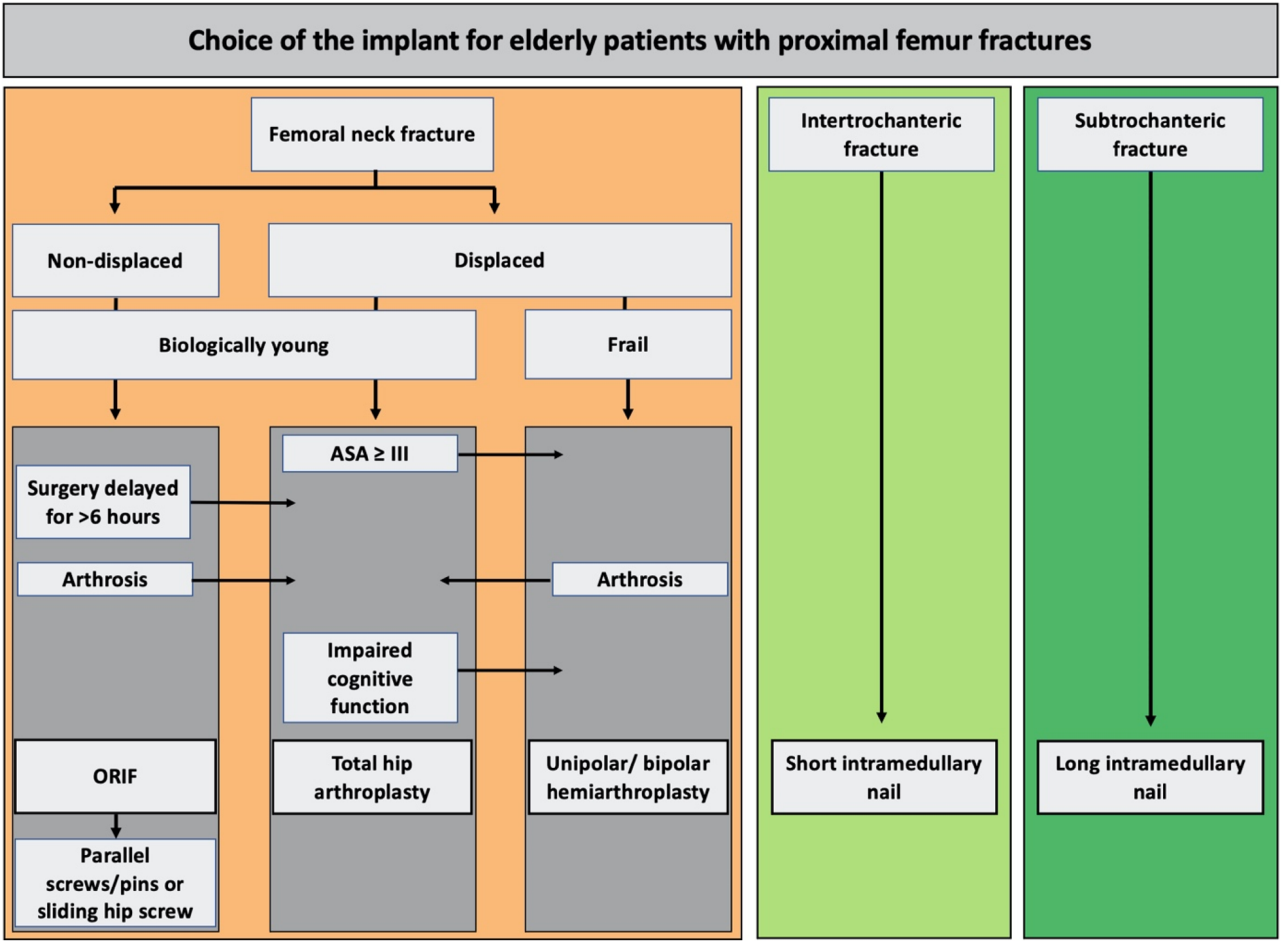
Choice of the implant in the operative treatment for femoral neck fractures in the elderly

**Fig. 5:**
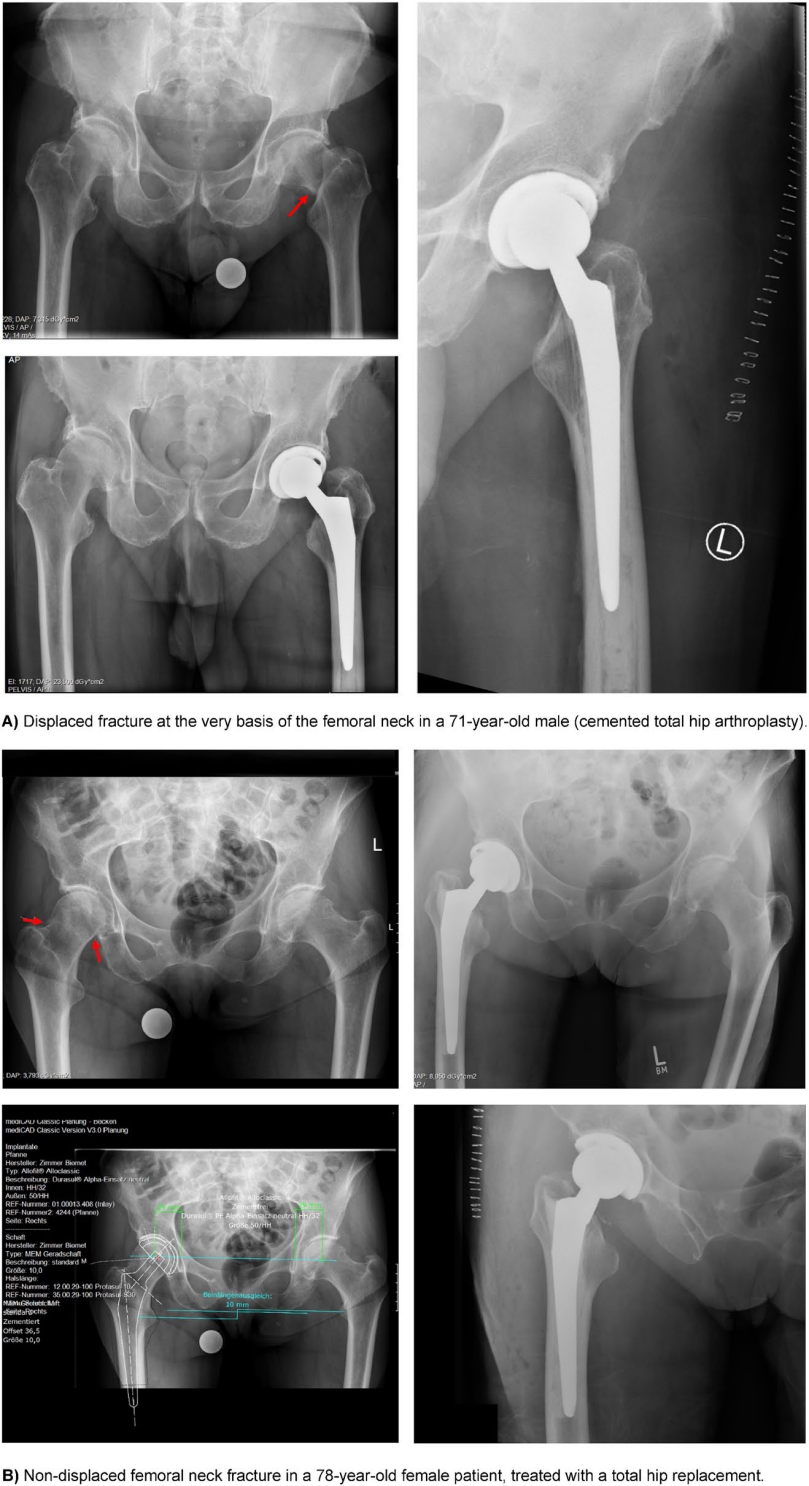

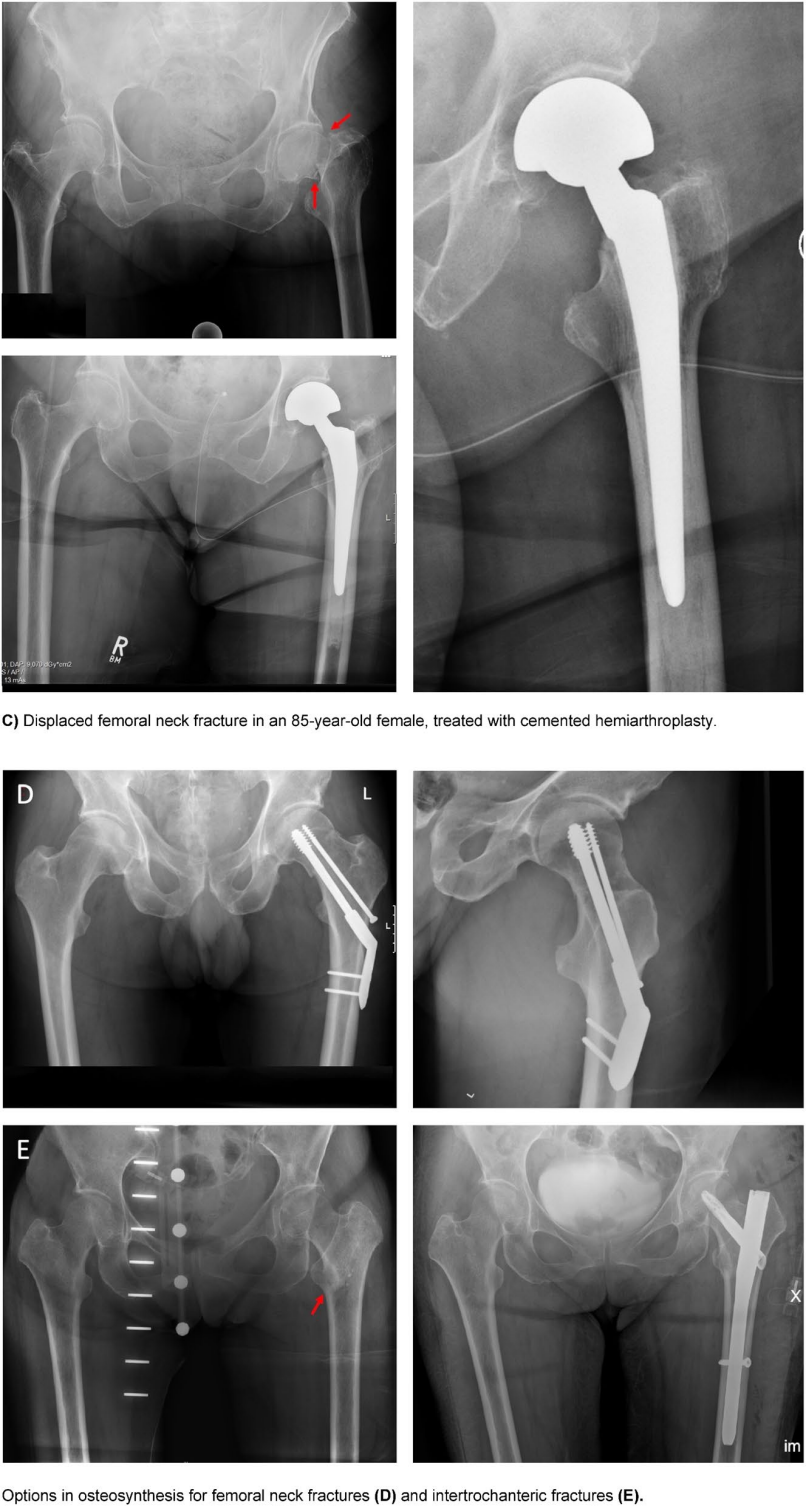
Different hip fractures and treatment options. **A** Displaced fracture at the very basis of the femoral neck in a 71-year-old male (cemented total hip arthroplasty). **B** Non-displaced femoral neck fracture in a 78-year-old female patient, treated with a total hip replacement. **C** Displaced femoral neck fracture in an 85-year-old female, treated with cemented hemiarthroplasty. Options in osteosynthesis for femoral neck fractures (**D**) and intertrochanteric fractures (**E**)

The surgical treatment should focus on the biological and not on the chronological age (Figs. 4 and 5) [30].

## Postoperatively

Patients benefit from early mobilisation, since the process reduces complication rates, and minimises the risk of pneumonia, thromboembolism, pressure ulcers, and delirium [68].

Patients with one fracture are at an increased risk of suffering another [69]. Therefore, it is essential to investigate the reasons for falling to prevent further fractures. Among the most common reasons are syncope, Parkinson’s disease and polypharmacy. Polypharmacy in general and drugs related to an increased risk of falling can present a preventable reason for fractures in the elderly [70].

Standard postoperative care should include mechanical thromboembolism prophylaxis such as early mobilisation, regular physiotherapy and pharmacological prophylaxis. According to Flevas et al., the use of low-molecular-weight heparin is preferable and should be continued for 28–35 days according to product characteristics (Table 3) [71].

**Table 3:** The big five in management of geriatric patients with femoral neck fractures (compiled from the AO-guidelines)

The big five in management of a geriatric patient with a femoral fracture to avoid the most common complications	
Time to surgery	The less time passes from admission to surgery, the fewer complications
Pain management	Pain management can be accomplished by a stable fixation, paracetamol, oral or parenteral opioids and regional nerve blockades
Delirium prevention	Prevention is the best strategy concerning delirium. Thorough fluid management (pre and postoperatively), help with orientation, avoiding of tethers such as tubes (urine catheter removal on the second day postoperatively if possible), help with orientation like for example hearing aids, proper pain management and hydration management contribute to lower the incidences of delirium
Early mobilisation	Physiotherapy and respiratory therapy prevent pneumonia and thrombotic events. Anticoagulation is needed for 28–35 days
Patient care	A proper postoperative bowel regimen prevents obstipation, pressure soars can be avoided by early surgery and frequent repositioning

## The impact of COVID-19 on hip fractures in the elderly

While the total number of fracture patients was significantly reduced during the COVID-19 pandemic globally, the number of fragility fractures remained stable [72].

Concerning COVID-19, most hip fracture patients comprise a high-risk population. Therefore, in COVID-19-negative patients, preventing COVID-19 infections in hospitals is of utter importance.

A systematic review and meta-analysis from Lim and Pranata reported a seven-fold increased risk of mortality for COVID-19-positive patients with hip fractures and, correspondingly, the risk of postoperative complications increased [73]. COVID-19-associated changes within the hospital led to additional challenges in medical care for elderly people. For example, waiting for COVID-19 tests, limited operating capacity, and the shortage of hospital staff in particular all affect both COVID-19-positive and COVID-19-negative patients. A Spanish multicentre study concerning the treatment of PFFs during the COVID-19 outbreak showed a mean delay of 2.4 days to surgery with a minimum of 0 days and a maximum of 13 days [74]. Also, data from Argentina confirmed a significantly prolonged time from admission to surgery during the COVID-19 pandemic in COVID-19-negative patients [75].

Cheung and Forsh stated that asymptomatic and mildly symptomatic COVID-19-positive patients with PFF might require preoperative medical optimisation, but that they can safely undergo early surgery. Both asymptomatic and mildly symptomatic COVID-19-positive patients might have an increased oxygen demand postoperatively [76].

## Conclusion

Providing medical care to elderly patients with hip fractures remains a great challenge. Interdisciplinary orthogeriatric management reduces the length of hospital stay, the number of complications and mortality.

The most critical peri-operative management aspects include proper pain management, early mobilisation, a thorough fluid management, the prevention of delirium and the choice of operative treatment depending on comorbidities, demands, and biological rather than chronological age. For elderly patients, direct weight bearing and as little delay as possible in operative treatment are of great importance. While inter- or subtrochanteric fracture requires intramedullary nailing, the treatment options for femoral neck fractures include osteosynthesis, total hip arthroplasty and hemiarthroplasty. The Garden classification and the patient’s activity level may allow osteosynthesis treatment for a biologically young patient with a non-dislocated fracture showing no signs of osteoarthritis. Total hip arthroplasty is recommended for active patients with dislocated fractures, and hemiarthroplasty for frail patients.

The COVID-19 pandemic brings additional obstacles in medical care for elderly hip fracture patients, leading to a delay in surgery, corresponding to a higher complication rate.

## Data Availability

Not applicable.
